# Supplementary material for: Study on the quantitative analysis of Tilianin based on Raman spectroscopy combined with deep learning
Source: PLoS One. 2025 Jun 18;20(6):e0325530. doi: 10.1371/journal.pone.0325530 (PMC12176167; doi:10.1371/journal.pone.0325530)
Supplement: Attachment — Submitted filename: Raman review.pdf [file pone.0325530.s001.pdf]

The manuscript presents an innovative approach to the quantitative analysis of Tilianin using Raman spectroscopy combined with deep learning. This work proposes an approach for Tilianin quantification and seems likely to compare the proposed deep learning model with other traditional and machine learning models; however, there are some weaknesses that should be addressed before being considered for publication.

1. The readability of the manuscript is rather low. Particularly, the introduction is poorly written. Please take a careful consideration to revise the text to improve the readability.
2. The authors are requested to cross check the references to ensure they are placed properly. For instance, in Introduction section, the reference 22 discusses CNN and not VAE. Please verify.
3. The last paragraph of Introduction is poorly written, and the novelties are very unclear. The authors need to spend some time to improve the quality of writing and describe all the contributions of the work clearly.
4. There are other works in the literature that have addressed similar challenges with either a greater number of data or data augmentation techniques. Some of these works are:

    a) Rashedi, M., Khodabandehlou, H., Wang, T., Demers, M., Tulsyan, A., Garvin, C., and Undey, C. (2024). Integration of just-in-time learning with variational autoencoder for cell culture process monitoring based on Raman spectroscopy. Biotechnol. Bioeng, 121, 1-20. doi:10.1002/bit.28713
    b) Min, R., Wang, Z., Zhuang, Y., and Yi, X. (2023). Application of semi-supervised convolutional neural network regression model based on data augmentation and process spectral labeling in Raman predictive modeling of cell culture processes. Biochemical Engineering Journal, 191(108774), ISSN 1369-703X.
    c) Khodabandehlou, H., Rashedi, M., Wang, T., Tulsyan, A., Schorner, G., Garvin, C., and Undey, C. (2024). Cell culture product quality attribute prediction using convolutional neural networks and raman spectroscopy. Biotechnol. Bioeng, 121, 1231-1243. doi:10.1002/bit.28646.

    The authors are requested to describe the contributions of their work compared to the mentioned references.

5. In many places in the text, the word “And” is used right after the end of a finished sentence. Please correct/remove them throughout the manuscript.
6. In section 2.1 it is written “Weigh the desired mass of Tilianin powder on a balance and transfer it to a beaker. Using a pipette measure less than the target volume of methanol into the beaker. Heat gently and stir well using a glass rod until Tilianin is completely dissolved. Transfer the dissolved Tilianin solution to a volumetric flask. Use a standard volumetric flask to finalize the volume and rinse the residual material several times to ensure that all Tilianin enters the solution system.” This type of writing is not a common practice in academic writing and needs a major revision. Please revise these sentences throughout the text accordingly.
7. In section 2.2, the authors are needed to explain how the offline measurements are labeled with the Raman spectra in the training dataset.
8. Section 2.3, de-meaned → detrended

9. In section 2.3 appropriate references are required for the baseline removal. Some of those references are:
   - Eilers, P., & Boelens, H. F. M. (2005). Baseline correction with asymmetric least squares smoothing (Leiden University Medical Centre Report).
   - Cai, Y., Yang, C., Xu, D., & Gui, W. (2018). Baseline correction for Raman spectra using penalized spline smoothing based on vector transformation. Analytical Methods, 10, 3525‑3533.

   Please consider providing comparison with these references to validate your approach.
10. In figure 1, the wave numbers are shown from $550\ cm^{-1}$ to $2000\ cm^{-1}$. Is this the ultimate range of wavenumbers? What happens to the spectra before or after this range? How many wavenumbers does the Raman machine provide?
11. In second paragraph of section 2.4, the authors have mentioned a classification model can be made between Raman spectra and concentrations. Do the authors mean a regression model? Otherwise, please clearly explain what the classes are in the classification. Then, please explain why classification is needed?
12. In the caption of figure 2, please type in what preprocessing steps are implemented on the Raman spectra.
13. In section 3.1, what do the authors mean by 1×1 convolutional channel compression? Please clarify. Also, please show where this is applied on Figure 4.
14. In the third paragraph of section 3.1, the authors discuss residual blocks. They say "the input features are directly added to a series of transformed outputs". They need to clarify which output they are talking about. I don't see any residual block for the output of the model in Figure 4.
15. The authors have tried to report the hyper parameters of the model structure in section 3.1, however, there are some missing details like the structure of fully connected layer.
16. In section 3.3 where the models are compared, a detailed description of all models is required. The competitive models lack detailed information regarding their hyper-parameters. Please revise this section appropriately.
17. Is this VAE model generated by the authors or has been used in other works? There are some curiosities about this model since a regenerated input in the decoder is used in fully connected layers. This provides a question why a regeneration of input improves the performance of compared with regular ANN. Please comment on this and clarify.
18. The authors are requested to discuss what specific challenges in Raman spectral analysis the proposed model is designed to overcome compared to prior models.
19. Why was SGD found to perform better than Adam? Could this be due to batch size or learning rate settings?
20. The self-attention mechanism is mentioned but not well explained in the context of spectral analysis. A brief mathematical or visual representation of how self-attention aids in feature extraction from Raman spectra would be beneficial.
21. The model performs well in lower concentration ranges but exhibits fluctuations at 0.2 mg/ml. What might be causing this? Is this due to saturation effects in Raman spectral intensities, or does the deep learning model struggle with higher concentration ranges?
22. While the paper shows strong performance metrics, a discussion on how RSAQN could be integrated into real pharmaceutical quality control workflows would be useful. Were any misclassification or prediction errors analyzed qualitatively? What types of spectra did the model struggle with?

23. Consider discussing potential applications beyond Tilianin (e.g., broader pharmaceutical compounds, food safety, biomedical diagnostics). Could transfer learning be applied to enhance model generalization across different Raman spectroscopy datasets?
