# Supplementary material for: Study on the quantitative analysis of Tilianin based on Raman spectroscopy combined with deep learning
Source: PLoS One. 2025 Jun 18;20(6):e0325530. doi: 10.1371/journal.pone.0325530 (PMC12176167; doi:10.1371/journal.pone.0325530)
Supplement: Attachment — Submitted filename: Response Letter.docx [file pone.0325530.s003.docx]

**Response Letter**

Firstly, we would like to thank you for your correspondence and the constructive feedback on our article. (Study on the quantitative analysis of Tilianin based on Raman spectroscopy combined with deep learning). These comments have been immensely valuable and have greatly assisted us in improving our article. All authors have thoroughly discussed these comments. In response to the editor's suggestions, we have made every effort to revise our manuscript to meet the requirements of your esteemed journal. In this revised version, changes to our manuscript have been highlighted in red text. Below is a point-by-point response to the editor and reviewers comments.

**Journal requirements:**

When submitting your revision, we need you to address these additional requirements.

1. Please ensure that your manuscript meets PLOS ONE's style requirements, including those for file naming. The PLOS ONE style templates can be found at

https://journals.plos.org/plosone/s/file?id=wjVg/PLOSOne_formatting_sample_main_body.pdf and

<https://journals.plos.org/plosone/s/file?id=ba62/PLOSOne_formatting_sample_title_authors_affiliations.pdf.>

**Response 1:**

We have ensured that our manuscript meets PLOS ONE's style requirements, including those for file naming.

2. Please note that PLOS ONE has specific guidelines on code sharing for submissions in which author-generated code underpins the findings in the manuscript. In these cases, we expect all author-generated code to be made available without restrictions upon publication of the work. Please review our guidelines at https://journals.plos.org/plosone/s/materials-and-software-sharing#loc-sharing-code and ensure that your code is shared in a way that follows best practice and facilitates reproducibility and reuse.

**Response 2:**

Our research cannot share code directly for legal or ethical reasons. We have stated this in our Data Availability Statement and can be contacted to request access to the code if needed.

3. We note that the grant information you provided in the ‘Funding Information’ and ‘Financial Disclosure’ sections do not match.When you resubmit, please ensure that you provide the correct grant numbers for the awards you received for your study in the ‘Funding Information’ section.

**Response 3:**

Our study was supported by [This work was sponsored by Natural Science Foundation of Xinjiang Uygur AutonomousRegion((2023D01C158), the "Tianshan Talents" Medical and Health High Level Talent Training Program Project (TSYC202301B068), and the Natural Science Foundation of Xinjiang Uygur Autonomous Region - General Project (2024D01C157)]. However, the "Tianshan Talents" Medical and Health High Level Talent Training Program Project (TSYC202301B068) is not normally retrieved and saved on the submission system, so it can only be shown in the reply letter.

4. Thank you for stating the following financial disclosure:[This work was sponsored by Natural Science Foundation of Xinjiang Uygur AutonomousRegion((2023D01C158), the "Tianshan Talents" Medical and Health High Level Talent Training Program Project (TSYC202301B068), and the Natural Science Foundation of Xinjiang Uygur Autonomous Region - General Project (2024D01C157)].

Please state what role the funders took in the study. If the funders had no role, please state: ""The funders had no role in study design, data collection and analysis, decision to publish, or preparation of the manuscript.""

If this statement is not correct you must amend it as needed.

Please include this amended Role of Funder statement in your cover letter; we will change the online submission form on your behalf.

**Response 4:**

The funders had no role in study design, data collection and analysis, decision to publish, or preparation of the manuscript. We have amended Role of Funder statement in our cover letter

5. Thank you for stating the following in the Acknowledgments Section of your manuscript:

[This work was sponsored by Natural Science Foundation of Xinjiang Uygur AutonomousRegion(2023D01C158), the "Tianshan Talents" Medical and Health High Level Talent Training Program Project (TSYC202301B068), and the Natural Science Foundation of Xinjiang Uygur Autonomous Region - General Project (2024D01C157)]

We note that you have provided funding information that is not currently declared in your Funding Statement. However, funding information should not appear in the Acknowledgments section or other areas of your manuscript. We will only publish funding information present in the Funding Statement section of the online submission form.

Please remove any funding-related text from the manuscript and let us know how you would like to update your Funding Statement. Currently, your Funding Statement reads as follows:

[This work was sponsored by Natural Science Foundation of Xinjiang Uygur AutonomousRegion((2023D01C158), the "Tianshan Talents" Medical and Health High Level Talent Training Program Project (TSYC202301B068), and the Natural Science Foundation of Xinjiang Uygur Autonomous Region - General Project (2024D01C157)].

Please include your amended statements within your cover letter; we will change the online submission form on your behalf.

**Response 5:**

Thank you for your suggestions to us. We have deleted the part of the text related to the funding in the manuscript and placed the funding information in the cover letter.

6. We note that you have indicated that there are restrictions to data sharing for this study. For studies involving human research participant data or other sensitive data, we encourage authors to share de-identified or anonymized data. However, when data cannot be publicly shared for ethical reasons, we allow authors to make their data sets available upon request. For information on unacceptable data access restrictions, please see http://journals.plos.org/plosone/s/data-availability#loc-unacceptable-data-access-restrictions.

Before we proceed with your manuscript, please address the following prompts:

a) If there are ethical or legal restrictions on sharing a de-identified data set, please explain them in detail (e.g., data contain potentially identifying or sensitive patient information, data are owned by a third-party organization, etc.) and who has imposed them (e.g., a Research Ethics Committee or Institutional Review Board, etc.). Please also provide contact information for a data access committee, ethics committee, or other institutional body to which data requests may be sent.

b) If there are no restrictions, please upload the minimal anonymized data set necessary to replicate your study findings to a stable, public repository and provide us with the relevant URLs, DOIs, or accession numbers. Please see http://www.bmj.com/content/340/bmj.c181.long for guidelines on how to de-identify and prepare clinical data for publication. For a list of recommended repositories, please see https://journals.plos.org/plosone/s/recommended-repositories. You also have the option of uploading the data as Supporting Information files, but we would recommend depositing data directly to a data repository if possible.

Please update your Data Availability statement in the submission form accordingly.

**Response 6:**

Thank you for recognizing our research, we have uploaded our research data as supplementary information，We also updated Data Availability statement in the submission form accordingly.

7. In the online submission form, you indicated that [The datasets generated and analyzed during the current study are not publicly available, but are available from the corresponding author upon reasonable request.].

All PLOS journals now require all data underlying the findings described in their manuscript to be freely available to other researchers, either 1. In a public repository, 2. Within the manuscript itself, or 3. Uploaded as supplementary information.

This policy applies to all data except where public deposition would breach compliance with the protocol approved by your research ethics board. If your data cannot be made publicly available for ethical or legal reasons (e.g., public availability would compromise patient privacy), please explain your reasons on resubmission and your exemption request will be escalated for approval.

**Response 7:**

Thank you for recognizing our research, we have uploaded our research data as supplementary information，We also updated Data Availability statement in the submission form accordingly.

8. Please include a copy of Table 2 which you refer to in your text on page 17.

[Note: HTML markup is below. Please do not edit.]

**Response 8:**

We have included below a copy of Table 2, which we cite in the text on page 17.

**Table 2 Comparison of performance with traditional machine learning and other deep learning models.**

|  | **R^2^** | **MSE** | **RMSE** | **MAE** |
| --- | --- | --- | --- | --- |
| **RF** | 0.6498 | 0.0012 | 0.0351 | 0.0318 |
| **KNN** | 0.8443 | 0.0005 | 0.0234 | 0.0212 |
| **ANN** | 0.8974 | 0.0003 | 0.0190 | 0.0147 |
| **CNN** | 0.8432 | 0.0005 | 0.0230 | 0.0187 |
| **VAE** | 0.8266 | 0.0005 | 0.0242 | 0.0178 |
| **RSAQN** | 0.9144 | 0.0003 | 0.0173 | 0.0136 |

**Reviewers' comments:**

**Reviewer #1:** The comments are attached as a pdf file, but the PLOS One website cannot accept the attachment only. So, I include all the comments so that I can proceed with submission. As a result, I request the authors to refer to the attachment to have a better understanding of the comments.

**Comment 1:**

The readability of the manuscript is rather low. Particularly, the introduction is poorly written. Please take a careful consideration to revise the text to improve the readability.

**Response 1:**

We sincerely thank the reviewers for their careful review of our introduction. We have completely rewritten the introduction section, optimizing both the article structure and language expression to significantly improve readability. All modifications have been clearly marked in red. Please refer to the revised manuscript for specific changes.

**Comment 2:**

The authors are requested to cross check the references to ensure they are placed properly. For instance, in Introduction section, the reference 22 discusses CNN and not VAE. Please verify.

**Response 2:**

We sincerely appreciate your careful review of our references. We apologize for any inaccuracies in the citations and have now thoroughly verified all references.

**Comment 3:**

The last paragraph of Introduction is poorly written, and the novelties are very unclear. The authors need to spend some time to improve the quality of writing and describe all the

contributions of the work clearly.

**Response 3:**

We sincerely appreciate your guidance regarding our introduction. Following the reviewers' comments, we have completely rewritten the concluding paragraph of the introduction to highlight the following key innovative contributions：

1. Propose the first customized attention residual network (RSAQN) for Tilianin, which effectively extracts spectral features at different levels through a multi-scale convolutional neural network architecture and self attention mechanism to achieve accurate feature peak recognition;

2.Significantly improve the accuracy and sensitivity of tilianin quantitative analysis compared to traditional methods.

The modified text has been highlighted in red and kindly reviewed.

**Comment 4:**

There are other works in the literature that have addressed similar challenges with either a greater number of data or data augmentation techniques. Some of these works are:

a) Rashedi, M., Khodabandehlou, H., Wang, T., Demers, M., Tulsyan, A., Garvin, C., and Undey, C. (2024). Integration of just-in-time learning with variational autoencoder for cell culture process monitoring based on Raman spectroscopy. Biotechnol. Bioeng, 121, 1-20. doi:10.1002/bit.28713

b) Min, R., Wang, Z., Zhuang, Y., and Yi,X. (2023). Application of semi-supervised convolutional neural network regression model based on data augmentation and process spectral labeling in Raman predictive modeling of cell culture processes. Biochemical Engineering Journal, 191(108774), ISSN 1369-703X.

c) Khodabandehlou, H., Rashedi, M., Wang, T., Tulsyan, A., Schorner, G., Garvin, C., and Undey, C. (2024). Cell culture product quality attribute prediction using convolutional neural networks and raman spectroscopy. Biotechnol. Bioeng, 121, 1231 -1243.doi:10.1002/bit.28646.

The authors are requested to describe the contributions of their work compared to the mentioned references.

**Response 4:**

We appreciate the reviewers for pointing out these representative related studies. We acknowledge that the aforementioned literature has made important contributions to Raman spectroscopy-based modeling and deep learning methods. However, our work demonstrates uniqueness and innovations in the following aspects:

The cited studies mainly focus on biopharmaceutical cell culture monitoring, typically relying on large-scale process Raman data. In contrast, our research specifically addresses the quantitative analysis of the natural drug active component Tilianin, particularly establishing models under small-sample and powdered-sample conditions. This application scenario is relatively rare in existing literature and presents challenges including subtle spectral variations and limited data availability.

Compared to previous approaches using complex architectures like variational autoencoders (VAE) and semi-supervised CNNs, our proposed custom network structure RSAQN offers the advantages of lightweight architecture and stable performance, making it particularly suitable for drug detection tasks with limited data. Additionally, we emphasize model interpretability, which is especially crucial in practical pharmaceutical analysis.

We have added comparative analyses with these references in Chapter 4 to further clarify the marginal contributions of this study.

**Comment 5:**

In many places in the text, the word “And” is used right after the end of a finished sentence. Please correct/remove them throughout the manuscript.

**Response 5:**

We sincerely appreciate the reviewers' guidance on grammatical conventions. We have conducted a systematic review of the entire manuscript and corrected all improper uses of sentence-initial 'And'.

**Comment 6:**

In section 2.1 it is written “Weigh the desired mass of Tilianin powder on a balance and transfer it to a beaker. Using a pipette measure less than the target volume of methanol into the beaker. Heat gently and stir well using a glass rod until Tilianin is completely dissolved. Transfer the dissolved Tilianin solution to a volumetric flask. Use a standard volumetric

flask to finalize the volume and rinse the residual material several times to ensure that all Tilianin enters the solution system.” This type of writing is not a common practice in

academic writing and needs a major revision. Please revise these sentences throughout the text accordingly.

**Response 6:**

We sincerely appreciate the reviewers' valuable suggestions regarding the methodological presentation. We have comprehensively revised Section 2.1 and other experimental description sections, transforming instructional language into objective academic discourse.

**Comment 7:**

In section 2.2, the authors are needed to explain how the offline measurements are labeled with the Raman spectra in the training dataset.

**Response 7:**

We appreciate your comments. The mean values from triplicate measurements were used as spectral labels to accurately determine Tilianin concentrations, providing ground truth references for the Raman spectral data. The relative standard deviation (RSD < 0.8%) was calculated through three repeated measurements to ensure data precision. The corresponding modifications have been highlighted in red in the manuscript.

**Comment 8:** Section 2.3, de-meaned detrended

**Response8 :** We sincerely appreciate the reviewers' precise suggestions regarding our grammar. We have implemented the corresponding revisions in Section 2.3.

**Comment 9:**

In section 2.3 appropriate references are required for the baseline removal. Some of those references are:

- Eilers, P., & Boelens, H. F. M. (2005). Baseline correction with asymmetric least squares smoothing (Leiden University Medical Centre Report).

- Cai,Y., Yang, C., Xu, D., & Gui,W. (2018). Baseline correction for Raman spectra using penalized spline smoothing based on vector transformation. Analytical Methods, 10, 3525-3533.

**Response 9:**

We sincerely appreciate the reviewers' recommended references and have incorporated them into Section 2.3

**Comment 10:**

In figure 1, the wave numbers are shown from 550 cm^−1^ to 2000 cm^−1^ . Is this the ultimate range of wavenumbers? What happens to the spectra before or after this range? How many wavenumbers does the Raman machine provide?

**Response 10:**

We sincerely appreciate the reviewers' valuable comments regarding the wavenumber range. Our final spectral range was determined as follows: while the Raman spectrometer's raw output covered 500-2000 cm^-1^ (1480 data points), Tilianin's characteristic peaks are predominantly distributed within 550-2000 cm^-1^(containing >95% of peak intensity). The sub-550 cm^-1^ region primarily contained silicon substrate signals and high-frequency noise, which were removed during preprocessing. Consequently, all figures display this optimized range.

**Comment 11:**

In second paragraph of section 2.4, the authors have mentioned a classification model can be made between Raman spectra and concentrations. Do the authors mean a regression model? Otherwise, please clearly explain what the classes are in the classification. Then, please explain why classification is needed?

**Response 11:** We sincerely appreciate the reviewers' precise positioning of our research problem. We confirm that this study employs regression models for concentration prediction, and agree that regression model is indeed a more accurate description.

**Comment 12:**

In the caption of figure 2, please type in what preprocessing steps are implemented on the Raman spectra.

**Response 12:**

We sincerely appreciate the reviewer's reminder. We have revised the caption of Figure 2 to: "Preprocessed Raman Spectra of Tilianin (airPLS baseline correction, SG smoothing, and normalization)."

**Comment 13:**

In section 3.1, what do the authors mean by 1 × 1 convolutional channel compression? Please clarify. Also, please show where this is applied on Figure 4.

**Response 13:**

We sincerely appreciate the reviewers' valuable suggestions. In Section 3.1, we have added the following clarification: The 1×1 convolutional channel compression reduces the 256-dimensional features to 64 dimensions through cross-channel linear combination (with ReLU activation), calculated as:

Z_out_ =ReLU(w_64x256_*Z_in_+b)

and have correspondingly updated Figure 4 to illustrate the 1×1 convolutional channel compression.

**Comment 14:**

In the third paragraph of section 3.1, the authors discuss residual blocks. They say “the input features are directly added to a series of transformed outputs”. They need to clarify which output they are talking about. I don’t see any residual block for the output of the model in Figure 4.

**Response 14:**

We sincerely appreciate the reviewers' comments and apologize for the oversight in our figure preparation. To clarify: our RSAQN model does not directly use residual blocks, but rather incorporates residual connection concepts to preserve original peak positions. The architecture employs multi-scale convolutional kernels to capture characteristic peaks and enhances feature representation through attention mechanisms. We have accordingly revised Figure 4.

**Comment 15:**

The authors have tried to report the hyper parameters of the model structure in section 3.1, however, there are some missing details like the structure of fully connected layer.

**Response 15:**

We sincerely appreciate the reviewers' valuable suggestions. We have supplemented the complete structural details of the fully connected layers in Section 3.1, with all modifications highlighted in red font in the revised manuscript.

**Comment 16:**

In section 3.3 where the models are compared, a detailed description of all models is required. The competitive models lack detailed information regarding their hyper-parameters. Please revise this section appropriately.

**Response 16:**

We sincerely appreciate the reviewers' valuable suggestions. In Section 3.3, we have comprehensively supplemented the hyperparameter details of all comparative models, including key parameters such as the number of trees/neighbors for traditional models (RF, KNN) and layer structures/channel numbers for deep learning models (CNN, VAE). These modifications have been highlighted in red in the revised manuscript.

**Comment 17:**

Is this VAE model generated by the authors or has been used in other works? There are some curiosities about this model since a regenerated input in the decoder is used in fully connected layers. This provides a question why a regeneration of input improves the performance of compared with regular ANN. Please comment on this and clarify.

**Response 17:**

We thank the reviewers for their in-depth attention to the VAE model design. The VAE architecture in this study is based on the classical framework by Kingma & Welling (2013), which we have now cited. The decoder output directly predicts concentration values rather than reconstructing original spectra, as drug concentration prediction is a regression task (not a generation task). This direct mapping from latent space to concentration values prevents information loss. Gradient backpropagation verification confirms that the encoded local characteristic peaks of Raman spectra in latent space retain essential quantitative information.

**Comment 18:**

The authors are requested to discuss what specific challenges in Raman spectral analysis the proposed model is designed to overcome compared to prior models.

**Response 18:**

We sincerely appreciate the reviewers' valuable suggestions. We have supplemented explanations in the Discussion section clarifying that our model aims to address three analytical challenges - low-concentration solution prediction, nonlinear effect correction at medium-high concentrations, and small-sample optimization design - through innovations including multi-scale feature extraction, adaptive attention mechanisms, and residual enhancement concepts. These modifications have been highlighted in red in Section 3.4.

**Comment 19:**

Why was SGD found to perform better than Adam? Could this be due to batch size or learning rate settings?

**Response 19:**

Thank you for your professional inquiry. To address this issue, we designed new supplementary experiments. In this experiment, we compared the performance of SGD (Stochastic Gradient Descent) and Adam (Adaptive Moment Estimation) optimizers under different configurations. To better understand the performance differences between these two optimizers, we adjusted the learning rates and batch sizes respectively, and conducted multiple sets of experiments. Below are the detailed analysis and validation results of the optimizers' performance.

**Table3：Comparison of SGD and Adam with Different Learning Rates**

| **Optimizer** | **Learning Rate** | **Batch Size** | **R²** | **RMSE** | **MAE** | **MSE** |
| --- | --- | --- | --- | --- | --- | --- |
| SGD | 0.01 | 32 | 0.91 | 0.0003 | 0.0173 | 0.00036 |
| SGD | 0.001 | 32 | 0.85 | 0.0225 | 0.0160 | 0.00040 |
| Adam | 0.01 | 32 | 0.87 | 0.0205 | 0.0152 | 0.00037 |
| Adam | 0.001 | 32 | 0.82 | 0.0243 | 0.0183 | 0.00045 |

**Table4：Batch Size Comparison**

| **Optimizer** | **Learning Rate** | **Batch Size** | **R²** | **RMSE** | **MAE** | **MSE** |
| --- | --- | --- | --- | --- | --- | --- |
| SGD | 0.01 | 32 | 0.91 | 0.0003 | 0.0173 | 0.00036 |
| SGD | 0.01 | 64 | 0.87 | 0.0212 | 0.0165 | 0.00040 |
| Adam | 0.01 | 32 | 0.87 | 0.0205 | 0.0152 | 0.00037 |
| Adam | 0.01 | 64 | 0.85 | 0.0221 | 0.0173 | 0.00041 |

The experimental comparison results demonstrate that Adam's adaptive learning rates for each parameter typically enable faster initial convergence during training, while SGD employs a fixed learning rate where its inherent noise helps escape local minima, contrasting with Adam's adaptive properties that may lead to premature convergence. Notably, SGD's implicit regularization synergizes with L2 weight decay, whereas Adam's adaptive learning rates may weaken explicit regularization effects, as evidenced by more pronounced oscillations in validation loss curves.

SGD conventionally utilizes smaller batch sizes, introducing stochastic noise in each parameter update that enhances training randomness and potentially facilitates escaping local optima, while Adam typically employs larger batches for more precise parameter updates. Across most configurations, SGD demonstrates superior stability - particularly when using smaller learning rates (0.001) where it achieves an R² of 0.91, significantly outperforming Adam's 0.82. Regarding batch size settings, SGD performs optimally at batch size 32 with slight degradation at 64, whereas Adam shows relatively poorer performance with larger batches (e.g., 64), likely because the increased batch size over-smooths optimization trajectories, causing Adam to more readily converge to local optima during solution space exploration.

**Comment 20:**

The self-attention mechanism is mentioned but not well explained in the context of spectral analysis. A brief mathematical or visual representation of how self-attention aids in feature extraction from Raman spectra would be beneficial.

**Response 20:**

We sincerely appreciate the reviewers' suggestions regarding our work. The incorporation of self-attention mechanisms enhances the model's ability to characterize Raman spectral features. As illustrated, the self-attention mechanism enables the model to learn relationships across all wavelengths rather than just local patterns. This proves particularly valuable for complex spectral data since Raman spectral correlations often extend beyond adjacent wavelengths. By computing inter-wavelength dependencies, the self-attention mechanism can identify global patterns including chemical component interactions and bond characteristics - features critical for Raman analysis. Furthermore, the mechanism dynamically focuses on different spectral regions during training, allowing automatic learning of wavelength features most relevant to specific analytical targets (e.g., component quantification or material identification). All supplementary analyses have been highlighted in red in the manuscript.

Figure 5 Self-Attention for Raman Spectroscopy feature extraction

**Comment 21:**

The model performs well in lower concentration ranges but exhibits fluctuations at 0.2mg/ml. What might be causing this? Is this due to saturation effects in Raman spectral intensities, or does the deep learning model struggle with higher concentration ranges?

**Response 21:**

We sincerely appreciate the reviewers' valuable comments regarding the prediction fluctuations at the 0.2 mg/mL concentration point. To further investigate potential causes, we analyzed the prediction error rates across all concentration points. Under consistent model training and testing conditions, the deviation rate (%) was calculated as:Deviation rate (%) = |Predicted value - Actual value| / Actual value × 100%, The obtained results are presented in the table as shown.

**Table5：Deviation Rate of Predicted Values**

| **Concentration (mg/mL)** | 0.2 | 0.1 | 0.08 | 0.06 | 0.04 | 0.02 |
| --- | --- | --- | --- | --- | --- | --- |
| **Average Predicted Values (mg/mL)** | 0.179 | 0.091 | 0.075 | 0.059 | 0.037 | 0.018 |
| **Deviation Rate** | 10% | 9% | 6% | 2% | 7% | 1% |

As shown in the charts, the deviation rate at the 0.2 mg/mL concentration point is indeed significantly higher than at other points. Two potential causes for this phenomenon include:

1.Raman spectral saturation effects: At intermediate concentrations (e.g., 0.2 mg/mL), Raman signals may approach saturation or exhibit band overlapping, resulting in less distinct features that increase the nonlinear fitting challenge for the model.

2.Model generalization and data distribution issues: The training samples at this concentration show greater variability, and deep learning models are highly sensitive to sample distribution, particularly when regularization or data augmentation strategies are insufficient.

Through further analysis, we observed that in lower concentration ranges, the absolute errors are relatively small, whereas in higher concentration ranges, the proportional errors relative to the sample concentration become more pronounced. In other words, higher concentrations tend to yield larger absolute errors. This explains why visual distinctions appear more significant when plotted together, despite the model maintaining an average deviation rate below 10% across all concentrations during training.

**Comment 22:**

While the paper shows strong performance metrics, a discussion on how RSAQN could be integrated into real pharmaceutical quality control workflows would be useful. Were any misclassification or prediction errors analyzed qualitatively? What types of spectra did the model struggle with?

**Response 22:** We sincerely appreciate your concern. We confirm that no qualitative analysis was conducted on incorrect classifications, and the model did not encounter the mentioned issues during spectral feature extraction in our experiments.

**Comment 23:**

Consider discussing potential applications beyond Tilianin (e.g., broader pharmaceutical compounds, food safety, biomedical diagnostics). Could transfer learning be applied to enhance model generalization across different Raman spectroscopy datasets?

**Response 23:**

We sincerely appreciate the reviewers' valuable suggestions. We believe the Raman spectroscopy-based deep learning modeling method proposed in this study possesses broad applicability that extends beyond Tilianin quantification. Its core concepts and workflow can be generalized to multiple domains, particularly for pharmaceutical quality control and identification. The model can be adapted for quantitative detection of other active components (e.g., flavonoids, saponins, alkylphenols, etc.), facilitating consistency evaluation of traditional Chinese medicine preparations. Our approach demonstrates excellent transferability and generalization potential.

**Reviewer #2:** This study presents a novel Raman-based deep learning model (RSAQN) for non-destructive Tilianin quantification. In contrast to conventional destructive HPLC/MS techniques, RSAQN employs self-attention mechanisms to analyze spectral features from six concentration levels (120 samples total). The model demonstrates superior performance over five benchmark ML/DL approaches, achieving an R² value of 0.9144, thereby enabling rapid and accurate drug quality assessment. However, several issues require attention:

**Comment 1:** The current English expression needs improvement - the descriptions are unnecessarily complex, sentences are overly long, and the overall organization lacks clarity. A more concise and straightforward presentation would significantly enhance readability.

**Response 1:**

We sincerely thank the reviewers for their valuable comments on our English expression. We have made systematic improvements to the full text, with a focus on enhancing the conciseness and readability of the expression. All changes have been highlighted in red in the revised manuscript. These changes have significantly improved readability while maintaining scientific rigor.

**Comment 2:** The Introduction section disproportionately focuses on HPLC, which is not the manuscript's primary focus. The HPLC discussion should be condensed, while more emphasis should be placed on Tilianin measurement techniques and relevant machine learning methods. A comparative analysis of key parameters would better highlight the advantages of the proposed method.

**Response 2:**

Thank you for your valuable suggestions on the introduction section. We have reduced the length of the HPLC discussion to retain only the necessary background and to focus more on Tilianin measurement techniques and related machine learning methods. In Section 3.3, we add details on the hyperparameters of all the compared models, including key parameters such as the number of trees/neighbors for the traditional models (RF, KNN), and the layer structure/number of channels for the deep learning models (CNN, VAE). And we have comparative analysis of key parameters for RSAQN in Table 4 and Table5. These modifications are highlighted in red in the revised manuscript.

**Comment 3:** In the Data Acquisition section: The characteristic peak at 520.7 cm^-1^ - which molecular component does this correspond to?

**Response 3: This peak does not originate from the Tilianin molecule, but rather represents a standard reference peak from the silicon wafer substrate used in the experiments. The silicon wafer substrate was selected due to its chemical inertness (non-reactive with Tilianin/methanol) and signal stability (RSD < 1%). The revised description has been highlighted in red in Section 2.2.**

**Comment 4:** For the Spectral Data Analysis section: Including a reference table assigning various Raman peaks would significantly improve data interpretation.

**Response 4:** Thank you for your valuable suggestions on the spectral data analysis section. We have added a new Supplementary Material, Table1, which lists the attribution of the main Raman characteristic peaks of field thistle glycosides and their significance for quantitative analysis.

**Comment 5:** In the Model Testing and Analysis section: The meaning of "fluctuations controlled within 0.02" is unclear - does this refer to an absolute value of 0.02 or 2% variation? Additionally, the claim regarding the model's accuracy and robustness in low-concentration ranges (based solely on Figure 2) requires stronger validation. Additional methods such as cross-validation and noise injection testing should be incorporated to substantiate these conclusions.

**Response 5:**

We appreciate your comments regarding the model testing analysis. We have clarified that the "0.02" refers to the absolute concentration value (mg/mL) rather than percentage fluctuation. The revised statement reads: "The predicted concentration fluctuation is controlled within ±0.02 mg/mL absolute concentration range."

We fully recognize the importance of validating the model's accuracy and robustness in low-concentration ranges, which is crucial for practical applications. In Section 3.4 Model Testing and Analysis, we have supplemented additional experiments to strengthen the validation of low-concentration accuracy through the following approaches:

1.Added 5-fold cross-validation experiments: Using stratified sampling to ensure each validation fold covers all concentration ranges, preventing evaluation bias due to sample imbalance.

2.To simulate real-world noise interference, we introduced Gaussian noise to the training data and performed repeated model training. This approach allowed us to evaluate the model's performance under both cross-validation and noise-injection testing conditions.

**Table 6：Experimental results of cross-validation and noise injection**

| **Verification method** | MSE | RMSE | MAE | R² |
| --- | --- | --- | --- | --- |
| **cross-validation** | 0.000380 | 0.0195 | 0.0151 | 0.911497 |
| **noise injection** | 0.000391 | 0.0198 | 0.0155 | 0.887597 |
| **Final test** | 0.000382 | 0.019546 | 0.015075 | 0.887269 |

These additional verification steps effectively enhance the reliability of the model and fully demonstrate its accuracy and robustness in the low concentration range. We have supplemented these verification results in the revised manuscript.

  Thank you to the editor and reviewers for giving us the opportunity to revise and improve. We have carefully optimized the article based on the comments and look forward to your review and further valuable suggestions.
